# Categorical perception in monkeys: modeling implicit learning of discrete categories

**DOI:** 10.1186/1471-2202-14-S1-P288

**Published:** 2013-07-08

**Authors:** Samarth Chandra, Mark Eldridge, Félix Hartmann, Narihisa Matsumoto, Barry Richmond, Jean-Pierre Nadal

**Affiliations:** 1 Laboratory of Neuropsychology, NIMH/NIH/DHHS, Bethesda, MD 20892-4415, USA; 2 Laboratoire de Physique Statistique, CNRS UMR8550, Ecole Normale Supérieure, Paris, 75231 cedex 05, France; 3 LERFoB, UMR INRA-AgroParisTech 1092, Centre INRA de Nancy-Lorraine, 54280 Champenoux, France; 4 Human Technology Research Institute, National Institute of Advanced Industrial Science and Technology, Tsukuba, Ibaraki 305-8561, Japan; 5 Centre d'Analyse et de Mathématique Sociales, CNRS UMR8557, Ecole des Hautes Etudes en Sciences Sociales, Paris, 75244 cedex 13, France

## 

Monkeys can learn discrete categories (such as 'cat' / 'dog') while performing a behavioral task without explicit instruction related to the categories [1]. The reward protocol is such that the monkey always gains some reward if it performs correctly an easy learnable task, but it can considerably increase its cumulative reward if it can make a proper use of categorical cues, which requires being able to distinguish between the two categories.

These experiments have not allowed to compare the acquired knowledge of categories with the standard phenomenon of *categorical perception *[2]. Notably, the main characteristic features of categorical perception show up in the psychophysics when the stimuli are ambiguous, near the boundary between categories in stimulus space. On the theoretical side, these features have been shown to emerge as byproduct of optimal neural coding, optimality being defined in terms of information content and Bayesian decision, when the task is to decide which category the stimulus belongs to (identification task) [3,4].

In new experiments we have focused on the *transition *between categories, controlling for the degree of ambiguity of the cue. The experimental protocol is otherwise similar to the one in [1]. We have modeled these behavioral experiments with a focus on learning. Our experiments indicate that the monkey acquires 'categorical perception' (results to be presented in detail elsewhere). The modeling shows that a reinforcement learning scheme [5,6] can reproduce the main behavioral results, and gives some insight on how categorical perception builds up through learning. The neural and behavioral properties in the model are qualitatively similar to those derived for an identification task assuming optimal coding. Quantitatively, the details depend on the reward protocol. The model parameters can be fitted to match the experimental behavioral results quantitatively.

In the model, the visual cues are encoded by a population of neurons with tuning curves sharing a single global shape (e. g. a sigmoid form), but with idiosyncratic parameters. This assembly feeds a decision layer, producing the behavioral choice function - the probability to make one of two possible choices given the cue. We study the optimal choice function resulting from maximizing the cumulative reward over all the tuning curves parameters. We demonstrate a universality property, namely, for a large enough population code, the same optimal behavioral choice function is obtained whatever the shape of the tuning curves (subject to some weak restrictions). We show an exact mathematical procedure to construct the optimal set of parameters for a large class of shapes of tuning curves. We believe the method is applicable to a wide range of neural models.
